# Altered Peripheral Immune Profiles in First-Episode, Drug-Free Patients With Schizophrenia: Response to Antipsychotic Medications

**DOI:** 10.3389/fmed.2021.757655

**Published:** 2021-11-24

**Authors:** Lei Chen, Wen-Hui Zheng, Yang Du, Xue-Song Li, Yun Yu, Hua Wang, Yong Cheng

**Affiliations:** ^1^NHC Key Laboratory of Birth Defects Research, Prevention and Treatment, Hunan Provincial Maternal and Child Health Care Hospital, Changsha, China; ^2^Key Laboratory of Ethnomedicine for Ministry of Education, Center on Translational Neuroscience, Minzu University of China, Beijing, China; ^3^The Third People's Hospital of Foshan, Guangdong, China

**Keywords:** cytokine, schizophrenia, first-episode, drug-free, antipsychotics, biomarker, growth factor

## Abstract

Previous research has demonstrated aberrations in the levels of inflammatory cytokines in patients with schizophrenia (SCZ), but most of the respective studies have tested a narrow set of inflammatory cytokines. Here, we aimed to analyze broad immune profiles in the peripheral blood of the first-episode drug-free (FEDF) patients with SCZ at baseline and after an 8-week treatment with atypical antipsychotics. Serum samples from 24 FEDF patients with SCZ and 25 healthy control (HC) subjects were tested using Luminex multiplex analysis for 30 cytokines, chemokines, and growth factors. Multiple comparison tests demonstrated that interleukin-2 (IL-2), IL-4, interferon-gamma (IFN-γ), monokine induced by IFN-γ, and granulocyte colony-stimulating factor (G-CSF) levels were significantly increased, whereas those of the epidermal growth factor were significantly decreased in the FEDF patients with SCZ. Moreover, the levels of the 6 dysregulated cytokines as well as those of 12 additional soluble factors in FEDF patients with SCZ were significantly decreased after 8 weeks of antipsychotic treatment. Furthermore, the transcription of *G-CSF* and *IFN-*γ was significantly increased in FEDF patients with SCZ when compared with controls, and *G-CSF* and *IFN-*γ mRNA levels were highly correlated with their respective protein concentrations. Receiver operating characteristic curves showed that G-CSF and IFN-γ had good performance in differentiating between FEDF patients with SCZ and HC subjects. Taken together, our data revealed that FEDF patients with SCZ were accompanied by a unique pattern of immune profile, and antipsychotic medications seemed to suppress the immune function in these patients, which could be used to develop novel targets for the diagnosis and treatment of SCZ.

## Introduction

Schizophrenia (SCZ) is a severe mental disorder that has profound effects on society and affects individuals and families (1, 2). The disease is characterized by hallucination, division of thinking, violent attacks, and delusion of victimization. It affects ~1% of the population worldwide, with a male/female ratio of 1.4:1 (3). Although great efforts have been made to understand the etiology of SCZ over the last several decades, the mechanism underlying the pathogenesis of this disease remains elusive. Previous research suggested that both genetic and epigenetic factors played roles in the onset and/or development of SCZ. For example, clinical and preclinical studies have indicated that miRNA dysregulation may play an etiological role in SCZ (4–7), and genome-wide significant association studies have identified hundreds of risk genes for SCZ (8–10). Additionally, genetic studies have shown a correlation between genes that regulate the immune system and risk of SCZ; and immune dysfunction may be involved in the pathogenesis of SCZ in some individuals (10, 11). Therefore, exploring the abnormalities in the immune system in patients with SCZ has attracted the attention of researchers in the field.

Cytokines, acting as cell signal transduction proteins or polypeptides, mediate and regulate immune responses and inflammation (12). Cytokines not only partially cross the blood-brain barrier and bind receptors on the neurons or glial cells in the brain, but also contribute to dopaminergic, noradrenergic, and serotonergic neurotransmission, which suggests that these molecules directly influence neuronal function and play a role in psychiatric disorders, including SCZ (13, 14). Indeed, studies have shown abnormal levels of cytokines, including interferon-γ (IFN-γ), tumor necrosis factor-α (TNF-α), interleukin (IL)-1β, IL-2, IL-4, and IL-6 in SCZ patients with SCZ. However, clinical data on cytokine levels in patients with SCZ have been inconsistent across studies, and most studies only analyzed a narrow set of inflammation-related factors in these patients (15–19). Therefore, the immune profiles in patients with SCZ remain unclear.

In this study, we aimed to evaluate immune profiles in the first-episode drug-free (FEDF) patients with SCZ. To this end, we analyzed 30 serum cytokines, chemokines, and growth factors in the peripheral blood of these patients in comparison with healthy control (HC) subjects using a Luminex platform and tested the effects of antipsychotic medication on the 30 inflammatory-related factors in these patients. We then measured the transcriptional expression of *IFN-*γ and granulocyte colony-stimulating factor *(G-CSF)* in FEDF patients with SCZ. We further explored the potential of the selected cytokines as biomarkers of SCZ.

## Materials and Methods

### Samples and Subjects

We recruited 24 FEDF patients with SCZ from the Third People's Hospital of Foshan. The patients were diagnosed according to the Diagnostic and Statistical Manual of Mental Disorders, Fifth Edition, using a diagnostic interview. Positive and Negative Syndrome Scale (PANSS) was used to assess the severity of SCZ. Twenty-five HC subjects were recruited through advertisements in the Third People's Hospital of Foshan and were recorded to have no evidence of psychiatric or physical illnesses evaluated by trained psychiatrists. Demographic and clinical characteristics are presented in Supplementary Table 1.

All participants provided written informed consent before inclusion in this study. The study protocol was approved by the Ethics Committee of the Third Hospital of Foshan, Foshan, China, and the experiments were conducted in accordance with the Declaration of Helsinki.

Peripheral blood was collected from the patients and HC subjects in the morning after overnight fasting, and the samples were collected at baseline and after 8 weeks of treatment with atypical antipsychotic medications in the SCZ patients (19 patients were followed up). For serum collection, the blood samples were placed at room temperature for 1 h to allow clotting, and the serum samples were obtained by centrifugation at 3,000 × g for 10 min. The samples were stored at −80°C until further analyses.

### Luminex Multiplex Analysis

We measured serum cytokines, chemokines, and growth factors in patients with SCZ and HC using the commercially available human cytokine/chemokine/growth factor magnetic bead panel (ThermoFisher Scientific, Waltham, MA, USA, Cytokine Human 30-Plex Panel) on a Luminex platform. These experiments were performed according to the protocol from the manufacturer.

### Total RNA Extraction and qRT-PCR

We extracted total RNA from total blood samples using TRIzol (Thermo Fisher, Waltham, MA, US), as described previously (20). Then, the mRNA was converted to cDNA using the 5 × Easy Quick RT Master Mix according to the manufacturer's instructions (Sangon Biotech, Shanghai, China). qRT-PCR was performed using a Light Cycler 96 Real-Time PCR Detection System (Roche, Basel, Switzerland). Primer sequences were as follows: *G-CSF* forward, 5′-GAGAAGCTGGTGAGTGAGTGT-3′; *G-CSF* reverse, 5′- ATTCCCAGTTCTTCCATCTGCT−3′; *IFN-*γ forward, 5′- TCAGCTCTGCATCGTTTTGG−3′; *IFN-*γ reverse, 5′- TTCTGTCACTCTCCTCTTTCCA−3′; *GAPDH* forward, 5′- AATGAATGGGCAGCCGTTAG−3′; *GAPDH* reverse, 5′- GGACTGTGGTCATGAGTCCT -3′.

### Statistical Analysis

We analyzed the data and created graphs using GraphPad Prism (La Jolla, CA, USA) and SPSS (Armonk, NY, USA). We assigned the cytokine values as half of the lower limit detection when samples were undetectable, and data from Luminex platform were log-transformed before statistical analysis. For the 30 analyzed cytokines, chemokines and growth factors, basic fibroblast growth factor-basic (FGF-2), IL-13, IL-6, IL-17A, granulocyte macrophage colony-stimulating factor (GM-CSF), IL-15, IL-5, IL-2, IL-7, IL-4 had more than 50% of values below the detection limit for the test. Statistical differences between FEDF patients with SCZ and controls were initially examined using unpaired *t*-test or unpaired *t*-test with Welch's correction (if the homogeneity of variance assumption was violated) (21), and then were adjusted by multiple comparison corrections-false discovery rate. Changes in cytokine levels in patients with SCZ at baseline and after 8 weeks of the follow-up were performed using a paired *t*-test, and then were adjusted by multiple comparison corrections-false discovery rate. Statistically significant differences in *G-CSF* or *IFN-*γ levels between FEDF patients with SCZ and controls were determined using an unpaired *t*-test with Welch's correction (homogeneity of variance assumption was violated, *p* < 0.05). Pearson's correlation analysis was performed to assess the correlation between mRNA and protein expression levels. Receiver operating characteristic (ROC) curves were used to evaluate the accuracy in serum cytokines for the differentiation between SCZ patients and HC subjects, and the area under the curve (AUC) was calculated to evaluate the accuracy of the test. Statistical significance was set at *P* < 0.05. ^***^P < 0.001, ^**^P < 0.01, ^*^P < 0.05.

## Results

### Cytokine, Chemokine, and Growth Factor Levels in FEDF Patients With SCZ

**Figure 1:**
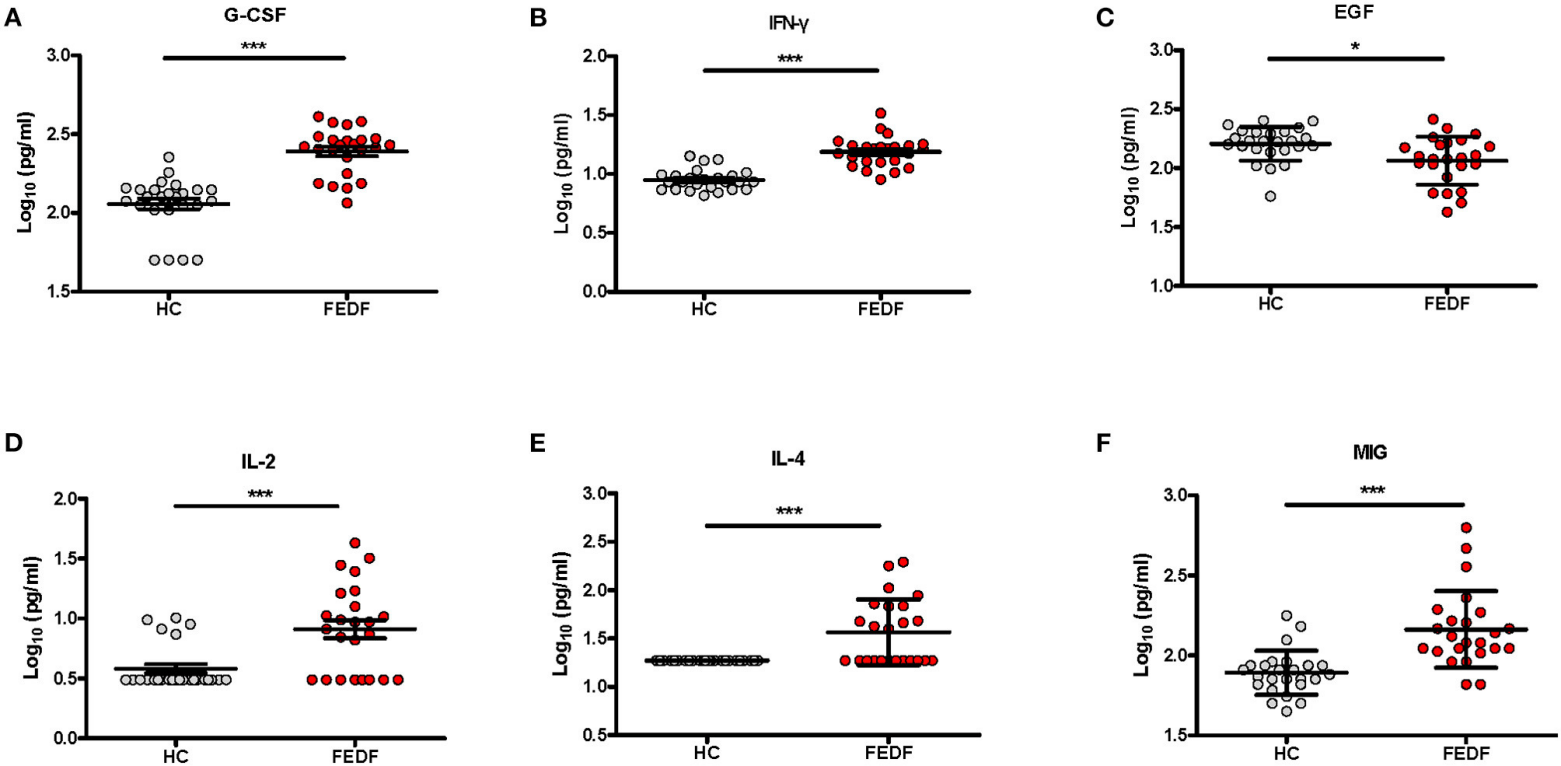
Serum levels of the G-CSF **(A)**, IFN-γ **(B)**, EGF **(C)**, IL-2 **(D)**, IL-4 **(E)**, and MIG **(F)** levels in FEDF patients with SCZ and HC subjects. SCZ, schizophrenia; FEDF, first-episode, drug-free; HC, healthy control; IFN, interferon; G-CSF, granulocyte colony-stimulating factors; MIG, monokine induced by interferon-γ; IL, interleukin; EGF, epidermal growth factor. *P < 0.05, ***P < 0.001.

In this study, we tested 30 cytokines using the Luminex platform in FEDF patients with SCZ and HC subjects. We found significantly higher levels of G-CSF (*P* < 0.001), IFN-γ (*P* < 0.001), monokine induced by interferon-γ (MIG, *P* < 0.001), IL-2 (*P* < 0.001), IL-4 (*P* < 0.001), IL-6 (*P* = 0.032), IL-7 (*P* = 0.034), IL-5 (*P* = 0.044), and hepatocyte growth factor (HGF, *P* = 0.039), and significantly lower levels of epidermal growth factor (EGF, *P* = 0.006) and macrophage inflammatory protein-1α (MIP-1α, *P* = 0.02) in FEDF patients with SCZ compared with the controls (Table 1). After multiple comparison corrections, G-CSF, IFN-γ, MIG, IL-2, IL-4, and EGF showed significant differences between cases and controls (Figure 1; Table 1).

**Table 1:** Dysregulated serum cytokine levels in FEDF patients.

	**HC (*n* = 25) (after Log_**10**_)**	**FEP (*n* = 24) (after Log_**10**_)**	**Homogeneity test of variance**		***P*-value**	***P*-value (adjust)**
			**F**	**Sig**		
G-CSF	2.057 ± 0.174	2.390 ± 0.147	0.63	0.803	3.88E-09	3.88E-08
IFN-γ	0.949 ± 0.088	1.188 ± 0.126	2.134	0.151	6.85E-10	1.03E-08
MIG	1.891 ± 0.138	2.162 ± 0.239	4.004	0.051	1.27E-05	9.53E-05
IL-2	0.581 ± 0.187	0.911 ± 0.367	10.227	0.002	3.86E-04	1.93E-03
IL-4	1.272	1.564 ± 0.340	82.067	<0.001	8.34E-05	5.00E-04
EGF	2.206 ± 0.143	2.062 ± 0.204	2.872	0.097	0.006	0.026
MIP-1α	1.909 ± 0.361	1.715 ± 0.156	22.493	<0.001	0.020	0.075
IL-7	1.346 ± 0.234	1.521 ± 0.317	10.847	0.002	0.034	0.102
HGF	2.376 ± 0.158	2.483 ± 0.194	4.067	0.049	0.039	0.106
IL-6	0.851 ± 0.322	0.696 ± 0.122	17.371	<0.001	0.032	0.107
IL-5	0.979	1.040 ± 0.140	29.459	<0.001	0.044	0.110
IL-13	1.141 ± 0.303	1.304 ± 0.294	0.521	0.474	0.062	0.116
FGF-2	0.926 ± 0.260	1.093 ± 0.342	4.875	0.032	0.061	0.122
IP10	0.871 ± 0.177	0.753 ± 0.250	1.389	0.244	0.061	0.131
MIP-1β	2.283 ± 0.202	2.152 ± 0.270	1.564	0.217	0.059	0.136
IFN-α	1.888 ± 0.141	1.805 ± 0.185	1.073	0.306	0.084	0.148
MCP-1	2.591 ± 0.142	2.516 ± 0.172	0.964	0.331	0.102	0.170
Eotaxin	1.779 ± 0.168	1.654 ± 0.332	9.621	0.003	0.109	0.172
IL-1RA	2.347 ± 0.167	2.424 ± 0.186	0.017	0.898	0.134	0.201
IL-2R	2.395 ± 0.239	2.487 ± 0.193	0.181	0.673	0.146	0.209
IL-1β	1.053 ± 0.444	1.231 ± 0.421	0.760	0.388	0.157	0.214
VEGF	0.219 ± 0.219	0.317 ± 0.280	1.128	0.294	0.178	0.223
IL-10	0.791 ± 0.369	0.910 ± 0.224	4.530	0.039	0.177	0.231
IL-15	1.429 ± 0.296	1.540 ± 0.443	3.241	0.078	0.306	0.367
IL-8	1.823 ± 0.317	1.744 ± 0.312	0.015	0.903	0.386	0.429
IL-12	2.158 ± 0.089	2.123 ± 0.172	4.096	0.049	0.378	0.436
TNF-α	0717 ± 0.195	0.721 ± 0.224	0.006	0.939	0.949	0.949
RANTES	3.449 ± 0.081	3.452 ± 0.079	0.165	0.678	0.902	0.966
GM-CSF	0.287 ± 0.345	0.280 ± 0.232	0.077	0.782	0.938	0.970
IL-17A	1.394	1.394	0.131	0.719	\	\

*FEDF, first-episode, drug-free; HC, healthy control; G-CSF, granulocyte colony-stimulating factors; IFN-γ, interferon gamma; MIG, monokine induced by interferon-γ; IL, interleukin; EGF, epidermal growth factor; MIP, macrophage inflammatory protein; HGF, hepatocyte growth factor; VEGF, vascular endothelial growth factor; IFN-α,interferon alpha; MCP-1, macrophage chemoattractant peptide-1; FGF-2, basic fibroblast growth factor; IL-1RA, interleukin-1 receptor antagonist; IP10, interferon-γ-inducible protein 10; GM-CSF, granulocyte macrophage colony-stimulating factor; TNF-α, tumor necrosis factor alpha; RANTES, regulated on activation, normal T cell expressed and secreted*.

### Serum Cytokine Concentrations After Antipsychotic Treatments

**Figure 2:**
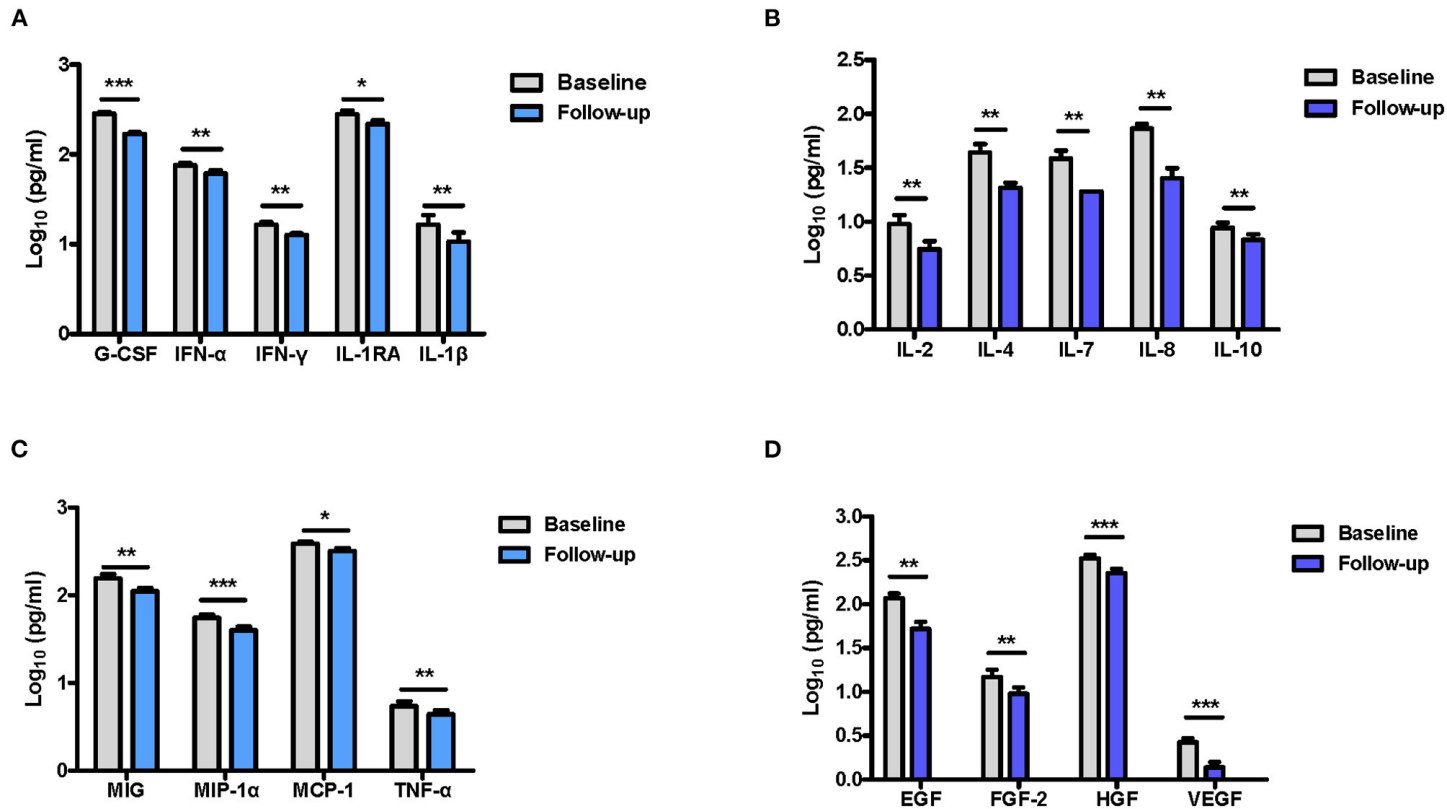
Differences in cytokine levels in FEDF patients with SCZ before and after treatment with antipsychotics. G-CSF, IFN-γ, IFN-α, IL-1RA, and IL-1β **(A)**, IL-2, IL-4, IL-7, IL-8, and IL-10 **(B)**, MIG, MIP-1α, TNF-α, and MCP-1 **(C)**, EGF, FGF-2, HGF, and VEGF **(D)** levels are significantly decreased in the serum of patients after 8 weeks of treatment with antipsychotic medication. SCZ, schizophrenia; FEDF, first-episode, drug-free; G-CSF, granulocyte colony-stimulating factors; IFN, interferon; MIG, monokine induced by interferon-γ; IL, interleukin; EGF, epidermal growth factor; MIP, macrophage inflammatory protein; HGF, hepatocyte growth factor; VEGF, vascular endothelial growth factor; MCP-1, macrophage chemoattractant peptide-1; FGF-2, basic fibroblast growth factor; TNF, tumor necrosis factor; IL-1RA, interleukin-1 receptor antagonist; *P < 0.05, **P < 0.01, ***P < 0.001.

To evaluate the antipsychotic medication effects on the blood cytokine levels in SCZ patients, we analyzed the differences in serum cytokine concentrations in FEDF SCZ patients between baseline and after an 8 week treatment with atypical antipsychotic medications. Of the 30 inflammatory-related factors tested using Luminex multiplex analysis, an 8 week treatment with atypical antipsychotics significantly reduced the levels of 18 cytokines, chemokines, and growth factors in the patients. These factors included the 6 cytokines that showed significant changes in levels between FEDF patients and controls and 12 additional soluble factors (Figure 2 and Supplementary Table 2). Therefore, these results suggest that atypical antipsychotics result in a systemic suppression of inflammation-related factors in FEDF patients with SCZ.

### Transcriptional Expression of *IFN-γ* and *G-CSF* in FEDF Patients With SCZ

**Figure 3:**
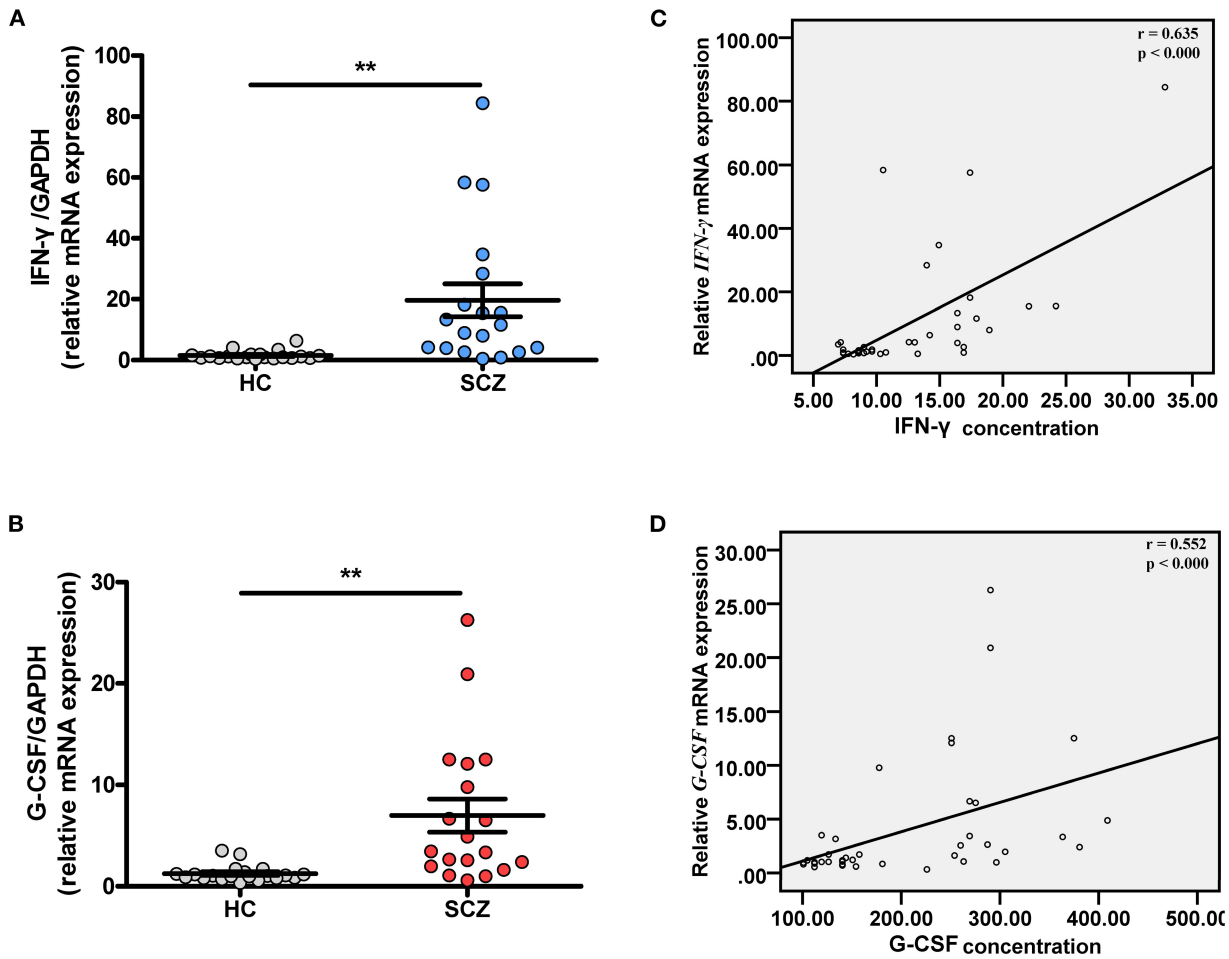
Transcriptional expressions of *G-CSF* and *IFN-*γ in FEDF patients with SCZ and HC subjects. Blood *IFN-*γ **(A)** and *G-CSF* **(B)** mRNA levels are significantly increased in FEDF patients with SCZ when compared with HC subjects. Pearson's correlation analysis shows that *IFN-*γ **(C)** and *G-CSF* **(D)** mRNA levels ware highly correlated with their respective protein concentrations. SCZ, schizophrenia; FEDF, first-episode, drug-free; HC, healthy control; IFN, interferon; G-CSF, granulocyte colony-stimulating factors; **P < 0.01.

We then explored the *G-CSF* and *IFN-*γ mRNA expression in the blood of FEDF patients using qRT-PCR, and the results suggested that *G-CSF* and *IFN-*γ mRNA levels were significantly higher in the blood of FEDF patients with SCZ than in the control subjects (unpaired *t*-test, *P* < 0.01, Figures 3A,B). Furthermore, significant positive correlations were found between mRNA expression and protein levels of G-CSF (r = 0.552, *P* < 0.001) and IFN-γ (*r* = 0.635, *P* < 0.001) (Figures 3C,D).

### IFN-γ and G-CSF as Diagnostic Biomarkers for SCZ

**Figure 4:**
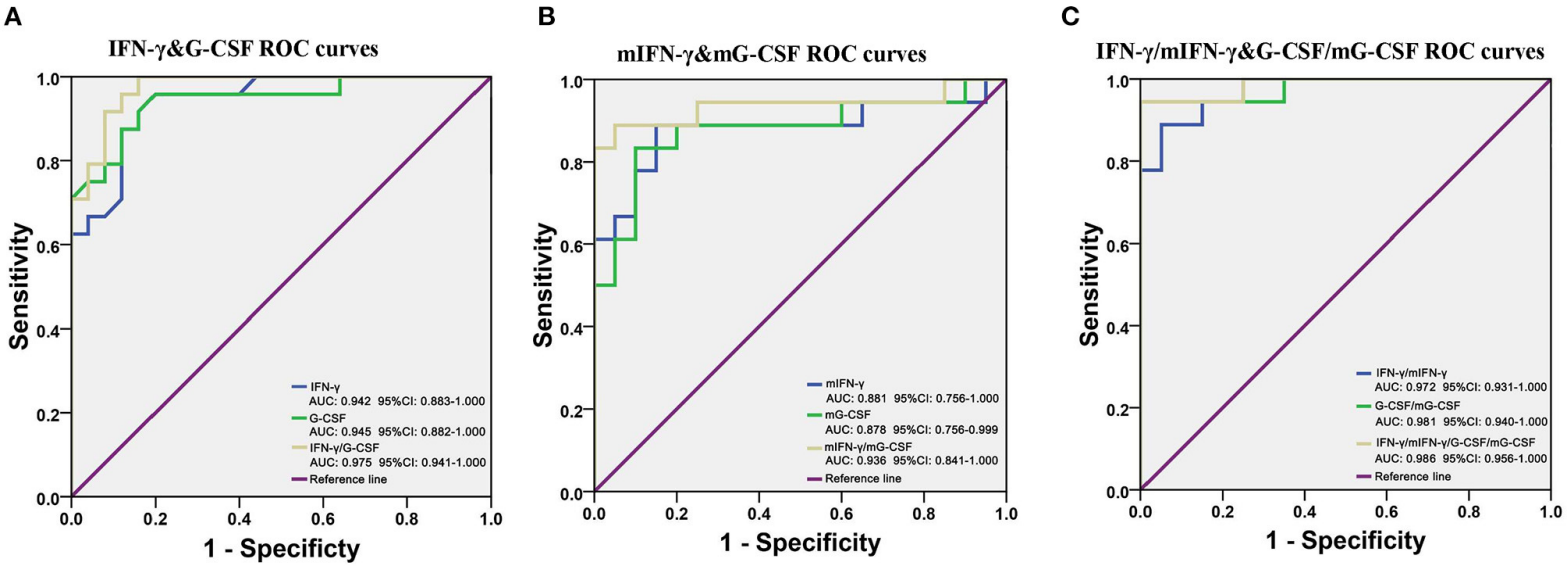
G-CSF and IFN-γ as potential biomarkers for SCZ. **(A)** ROC curves are utilized to evaluate the accuracy of the serum *G-CSF* and *IFN-*γ levels for differentiating between FEDF patients with SCZ and HC subjects. **(B)** ROC curves are utilized to evaluate the accuracy of blood *G-CSF* and *IFN-*γ mRNA levels for differentiating between FEDF patients with SCZ and HC subjects. **(C)** ROC curves are utilized to evaluate the accuracy of combining cytokine mRNA and protein levels for differentiating between FEDF patients with SCZ and HC subjects. Receiver operating characteristic (ROC) curves were used to evaluate the accuracy in serum cytokines for the differentiation between SCZ patients and HC subjects, and the area under the curve (AUC) was calculated to evaluate the accuracy of the test. SCZ, schizophrenia; ROC, receiver operating characteristic; FEDF, first-episode, drug-free; HC, healthy control; IFN, interferon; G-CSF, granulocyte colony-stimulating factors.

Given that IFN-γ and G-CSF were robustly and significantly dysregulated in FEDF patients with SCZ, ROC curves were used to evaluate the accuracy of IFN-γ and G-CSF in differentiating between FEDF patients and controls. On the basis of the ROC curve analysis, the optimal cutoff value of the serum G-CSF level to differentiate patients with SCZ was projected to be 142.0 pg/mL with a sensitivity of 95.8% and specificity of 80.0%, and an AUC of 0.945 [95% confidence interval (CI): 0.882-1.000]. The optimal cutoff value of the serum IFN-γ level for differentiating patients with SCZ was projected to be 10.08 pg/ml, which yielded a sensitivity of 95.8%, specificity of 80.0%, and AUC of 0.942 (95% CI: 0.883-1.000). Combining the values for IFN-γ and G-CSF increased the accuracy in differentiating between cases and controls, with an AUC of 0.975 (95% CI: 0.941-1.000), as shown in Figure 4A. Moreover, the transcriptional levels of *IFN-*γ *and G-CSF* also showed good performance in differentiating between FEDF patients with SCZ and HC subjects (Figure 4B). Additionally, combining protein and mRNA expression levels using ROC curves increased the accuracy of IFN-γ and/or G-CSF in diagnosing SCZ (Figure 4C). These results suggest that IFN-γ and G-CSF have the potential to inform the differentiation of SCZ.

## Discussion

In this study, we examined the peripheral immune profile of FEDF patients with SCZ before and after treatment with atypical antipsychotics. A unique serum profile that included a widespread characterization of the inflammatory signaling pathway was revealed in FEDF patients with SCZ. We found higher levels of G-CSF, IFN-γ, MIG, IL-2, IL-4, HGF, and IL-5, and lower EGF levels in FEDF patients with SCZ compared with HC subjects. *G-CSF* and *IFN-*γ mRNA levels were also increased in EFDF patients with SCZ when compared with controls, and *G-CSF* and *IFN-*γ transcriptional expression levels were highly correlated with their respective protein levels. Furthermore, an 8-week antipsychotic treatment significantly reduced the levels of 18 inflammation-related factors in the patients. Additionally, G-CSF and IFN-γ had good performance in differentiating between FEDF patients and HC subjects, suggesting the potential of these two inflammatory cytokines as biomarkers for the diagnosis of SCZ. To the best of our knowledge, this is the first study to analyze a broad panel of cytokines in FEDF patients with SCZ at baseline and follow-up with antipsychotic treatments. A previous study used the Luminex platform to evaluate a broad panel of cytokines in first-episode psychosis patients with SCZ, but it did not find significant differences in cytokine levels between the cases and controls (15). However, most of the patients were under antipsychotic medications at the time of testing in that study, which is likely to explain the observed difference between the two studies. Taken together, our results significantly enhanced our knowledge on the state of the peripheral inflammation in SCZ and treatment response to antipsychotics, and provided information that may be useful for the diagnosis of SCZ.

A noticeable finding in this study is that serum IFN-γ levels were robustly and significantly increased in FEDF patients with SCZ. In agreement with this finding, the cytokine MIG level was upregulated in patients with SCZ. This is generally consistent with previous meta-analyses showing increased blood IFN-γ levels in patients with SCZ, although high levels of between-study heterogeneities were found in these meta-analyses (16, 17). This discrepancy may largely be due to the antipsychotic medication effects, and this is supported by our data showing that an 8 week of antipsychotic treatment significantly reduced the blood levels of IFN-γ in the patients. Additionally, our data from the Luminex platform revealed that levels of another pro-inflammatory IL-2 were also upregulated in FEDF patients with SCZ. The increased levels of IFN-γ and IL-2 suggested an increase in Th-1 response in patients with SCZ, since IFN-γ and IL-2 are the major cytokines produced by Th-1 cells. The functional involvement of a pro-inflammatory response in the pathogenesis of SCZ was supported by a study suggesting that blocking stress-induced inflammatory responses by minocycline prevented behavioral abnormalities in an animal model of SCZ (22). The increased Th-1 response in the peripheral blood of SCZ patients may have an implication in the central nervous system, given that a post-mortem study showed significantly increased IL-2 and IL-12p70 levels in the hippocampus of patients with SCZ (23). Harris et al. analyzed 35 post-mortem SCZ patient samples and 33 controls and demonstrated higher levels of IFN-γ in patients compared with controls (24). Therefore, these results indicate the important role of the Th-1 response in the onset and/or development of SCZ and may provide therapeutic targets for alleviating symptoms in patients with SCZ.

In addition to the increase in the Th-1 response, we observed activation of the anti-inflammatory Th-2 system in patients with SCZ, as demonstrated by the increased IL-4 and IL-5 levels in these patients, although the statistical significance was lost for IL-5 after multiple comparison corrections. Additionally, for the first time, we have discovered that the levels of an anti-inflammatory growth factor, G-CSF, were significantly increased in FEDF patients with SCZ. Therefore, our data suggest that patients with SCZ at an early stage and prior antipsychotic medication presented both pro-and anti-inflammatory responses, although it is unclear which one had a greater response. Previous studies have largely focused on the pro-inflammatory response in SCZ (16–18, 24, 25), possibly because of the limited number of cytokines analyzed in patient samples; thus, the understanding of the immune response in patients with SCZ is limited. Our data highlight a potentially important role of the anti-inflammatory response in patients with SCZ. A possible explanation for this phenomenon is a self-defensive mechanism of the body under stress at the early stage of disease; therefore, future studies are necessary to evaluate the role of anti-inflammatory responses in SCZ.

Antipsychotic treatments are generally considered to have an anti-inflammatory effect in patients with SCZ, since the evidence including a meta-analysis suggested that antipsychotic treatment reduced pro-inflammatory cytokine levels, and therefore these drugs were thought to normalize immune balance dysfunction in SCZ (19). One interesting finding in this study is that antipsychotic treatment in the patients caused an overall reduction in blood cytokine levels, including classical pro-and anti-inflammatory cytokines and inflammatory-related growth factors. Growth factors including HGF, EGF, G-CSF, and FGF-2 also possess neurotrophic and/or neurogenic properties of the nervous system (26–29). These results suggest a detrimental effect of antipsychotics in patients, and may partially explain the observed brain structural changes in patients with SCZ after antipsychotic treatment (30). Additionally, EGF and the pro-inflammatory cytokine MIP-1α were downregulated in patients with FEDF, and antipsychotic treatment further decreased their levels. These results indicated that the effect of antipsychotic treatment was unlikely to normalize immune balance dysfunction in SCZ, but rather a systemic suppression of immune function, at least at the early stage of the disease. Thus, a broad panel of cytokine analysis performed in this study provided a cytokine change profile after antipsychotic medications in FEDF patients and highlighted the detrimental effect of antipsychotics on the immune system, which may partially contribute to the side effects of antipsychotic medications.

In addition to a better understanding of the cytokine profile before and after treatment in FEDF patients, this study has implications for developing novel biomarkers of SCZ. Biomarkers can not only explore the pathogenesis of the disease at the molecular level, but also have unique advantages in accurately and sensitively evaluating the early phase of SCZ. Although the diagnosis of SCZ is still made by lengthy subjective evaluation by clinicians, researchers have discovered various potential biomarkers that could inform the diagnosis and treatment response for SCZ. These potential biomarkers include brain-derived neurotrophic factor and nerve growth factor, and meta-analyses have demonstrated reduced peripheral blood levels in patients with SCZ and/or responsive to antipsychotics (31, 32). We have recently found a cluster of serum exosome-derived miRNAs that had reasonable performance in differentiating between FEDF patients and controls in two sets of participants, suggesting the potential of these miRNAs to inform the diagnosis of SCZ (6). Interestingly, the top differentially expressed exosomal miRNA in SCZ patients was miR-206, which has been shown to regulate the expression of brain-derived neurotrophic factor. Furthermore, cytokines have also been proposed as biomarkers for SCZ (33), but previous clinical studies rarely assessed the accuracy of cytokines in differentiating between cases and controls. Here, we used ROC curves to evaluate the potential in measuring the levels of the two cytokines IFN-γ and G-CSF, that were changing robustly, as biomarkers for SCZ. Both IFN-γ and G-CSF could differentiate between FEDF patients with SCZ and controls, with reasonable specificity and sensitivity, suggesting the usefulness of cytokines as biomarkers for the diagnosis of SCZ. However, there is an increasing awareness that combining multiple biomarkers reflective of different molecular pathways underlying the pathogenesis of SCZ may lead to a better and more accurate diagnosis of SCZ. In support of this hypothesis, the combination of IFN-γ and G-CSF proteins and/or mRNAs increased the accuracy of cytokines to differentiate between patients and controls. Therefore, future investigations with appropriate study designs are necessary to better evaluate the potential of SCZ-associated molecules as diagnostic and/or prognostic biomarkers.

In conclusion, our results add to the growing literature elaborating on the immune profile in SCZ using the Luminex technique. We observed a variety of dysregulated inflammatory cytokines, chemokines, and inflammatory-related growth factors in FEDF patients with SCZ. Furthermore, after 8 weeks of treatment with antipsychotic medication we found an overall reduction of inflammatory-related factors in these patients. Therefore, these findings could not support an anti-inflammatory mechanism of antipsychotics action in SCZ. Further analyses revealed that the two cytokines, G-CSF and IFN-γ, whose levels were robustly and significantly altered, had the potential to support a diagnosis of SCZ. Thus, this study provided a comprehensive within-subject cytokine profile in FEDF patients with SCZ before and after treatment with atypical antipsychotics, and provided potential targets for the diagnosis and treatment of this disease. However, one limitation of this study is that the sample size is relatively small. Nevertheless, future studies with large sample sizes in well-characterized populations are necessary to draw more definitive conclusions regarding the role of inflammation in SCZ.

## Data Availability Statement

The original contributions presented in the study are included in the article/Supplementary Material, further inquiries can be directed to the corresponding author/s.

## Ethics Statement

The studies involving human participants were reviewed and approved by the Ethics Committee at The Third Hospital of Foshan, Foshan, China. The patients/participants provided their written informed consent to participate in this study.

## Author Contributions

YC conceived and designed the study. LC, YD, and YY performed the experiments. LC, W-HZ, X-SL, and YC analyzed and interpreted the data. LC drafted the manuscript with critical revisions from YC and HW. All authors contributed to the article and approved the submitted version.

## Funding

This work was supported by the National Natural Science Foundation of China (82071676, 81703492), Beijing Natural Science Foundation (7182092), the Minzu University Research Fund (2018CXTD03), and the MUC 111 Project.

## Conflict of Interest

There is a patent pending application related to the results in the paper. The authors declare that the research was conducted in the absence of any commercial or financial relationships that could be construed as a potential conflict of interest.

## Publisher's Note

All claims expressed in this article are solely those of the authors and do not necessarily represent those of their affiliated organizations, or those of the publisher, the editors and the reviewers. Any product that may be evaluated in this article, or claim that may be made by its manufacturer, is not guaranteed or endorsed by the publisher.
