# Supplementary material for: Altered Peripheral Immune Profiles in First-Episode, Drug-Free Patients With Schizophrenia: Response to Antipsychotic Medications
Source: Front Med (Lausanne). 2021 Nov 24;8:757655. doi: 10.3389/fmed.2021.757655 (PMC8652082; doi:10.3389/fmed.2021.757655)
Supplement: Supplementary file 1 [file Data_Sheet_1.PDF]

**Supplement Table 1 Demographic and clinical characteristics of patients and controls**

| | HC (n=25) | FEDF(n=24) | Follow-up(n=19) |
|---|---|---|---|
| Age(years) | 27.00±5.132 | 26.46±6.487 | 27.10±5.955 |
| Male(%) | 40.00 | 54.17 | 47.37 |
| PANSS total score | / | 87.33±18.88 | / |
| PANSS positive score | / | 17.88±6.469 | / |
| PANSS negative score | / | 37.46±10.99 | / |
| Medication | / | / | Clozapine Tablets (20-450mg)<br>Aripiprazole (5-200mg)<br>Perphenazine Tablets (16-28mg)<br>OLanzapine Tablets (5-200mg)<br>Chlorpromazine (100mg)<br>Sulfapride tablets (200-800mg)<br>Risperidone tablets (2-6mg) |

HC, healthy control; FEDF, first-episode, drug-free.

**Supplement Table 2 Demographic and clinical characteristics of FEDF patients and Follow-up patients**

| | FEDF(n=19) (after $Log_{10}$) | Follow-up(n=19) (after $Log_{10}$) | P value | P value (adjust) |
|---|---|---|---|---|
| G-CSF | 2.453±0.086 | 2.229±0.082 | 4.23E-07 | 6.35E-06 |
| VEGF | 0.426±0.200 | 0.138±0.271 | 3.00E-05 | 3.00E-04 |
| HGF | 2.518±0.190 | 2.355±0.202 | 1.61E-04 | 9.64E-04 |
| MIP-1α | 1.745±0.154 | 1.601±0.187 | 3.29E-05 | 2.47E-04 |
| IL-4 | 1.641±0.343 | 1.316±0.192 | 2.10E-04 | 1.05E-03 |
| TNF-α | 0.737±0.233 | 0.643±0.213 | 2.89E-04 | 1.24E-03 |
| IL-1β | 1.217±0.464 | 1.028±0.449 | 4.02E-04 | 1.34E-03 |
| IL-8 | 1.863±0.195 | 1.403±0.411 | 3.82E-04 | 1.43E-03 |
| IL-7 | 1.585±0.328 | 1.281 | 0.001 | 0.003 |
| IFN-γ | 1.220±0.118 | 1.101±0.869 | 0.001 | 0.003 |
| IL-2 | 0.978±0.370 | 0.745±324 | 0.002 | 0.005 |
| EGF | 2.070±0.228 | 1.720±0.338 | 0.002 | 0.005 |
| FGF-2 | 1.172±0.343 | 0.979±0.312 | 0.003 | 0.006 |
| IL-10 | 0.942±0.206 | 0.832±0.227 | 0.003 | 0.006 |
| IFN-α | 1.878±0.113 | 1.789±0.166 | 0.005 | 0.009 |
| MIG | 2.191±0.221 | 2.047±0.163 | 0.006 | 0.010 |
| IL-1RA | 2.447±0.172 | 2.338±0.177 | 0.006 | 0.011 |
| MCP-1 | 2.589±0.096 | 2.503±0.154 | 0.009 | 0.014 |
| IL-5 | 1.056±0.154 | 0.979 | 0.042 | 0.063 |
| Eotaxin | 1.767±0.271 | 1.658±0.312 | 0.057 | 0.081 |
| IL-13 | 1.285±0.286 | 1.176±0.224 | 0.132 | 0.180 |
| GM-CSF | 0.302±0.257 | 0.235±0.171 | 0.162 | 0.211 |
| MIP-1β | 2.179±0.274 | 2.126±0.186 | 0.233 | 0.291 |
| IL-2R | 2.482±0.202 | 2.509±0.198 | 0.406 | 0.487 |
| IP10 | 0.804±0.202 | 0.839±0.226 | 0.609 | 0.703 |
| IL-15 | 1.549±0.478 | 1.522±0.447 | 0.642 | 0.713 |
| RANTES | 3.434±0.076 | 3.436±0.065 | 0.868 | 0.930 |
| IL-12 | 2.196±0.091 | 2.195±0.179 | 0.968 | 0.977 |
| IL-6 | 0.707±0.136 | 0.709±0.142 | 0.977 | 0.977 |
| IL-17A | 1.395 | 1.395 | \ | \ |

FEDF, first-episode, drug-free; G-CSF, granulocyte colony-stimulating factors; VEGF, vascular endothelial growth factor; IL, interleukin; HGF, hepatocyte growth factor; EGF, epidermal growth factor; MIP, macrophage inflammatory protein; IFN-γ, interferon gamma; FGF-2, basic fibroblast growth factor; IFN-α, interferon alpha; MCP-1, macrophage chemoattractant peptide-1; IL-1RA, interleukin-1 receptor antagonist; MIG, monokine induced by interferon-γ; TNF-α, tumor necrosis factor alpha; GM-CSF, granulocyte macrophage colony-stimulating factor; IP10, interferon-γ-inducible protein 10; RANTES, regulated on activation, normal T cell expressed and secreted.
